# Severe Coronary Vasospasms in Asymptomatic COVID-19

**DOI:** 10.7759/cureus.64869

**Published:** 2024-07-18

**Authors:** Omar M Masarweh, Ubaldo Gonzalez-Morales, Sara Tahir, Sana Tahir, Sayed T Hussain

**Affiliations:** 1 Internal Medicine, University of Central Florida, Orlando, USA; 2 Physiology, University of Florida, Gainseville, USA; 3 College of Science, University of Central Florida, Orlando, USA; 4 Cardiology, Heart and Vascular Institute, Osceola Regional Medical Center/University of Central Florida College of Medicine, Kissimmee, USA

**Keywords:** left heart catheterization, cardiology, st-elevation myocardial infarction (stemi), coronary artery vasospasm, covid 19

## Abstract

Since the emergence of the severe acute respiratory syndrome coronavirus 2 (SARS-CoV-2) in 2019, we have witnessed its multi-organ system involvement, not limiting itself to the lungs. We present a case of a patient with asymptomatic coronavirus disease 2019 (COVID-19) infection who developed ST-elevation myocardial infarction (STEMI) due to coronary artery vasospasm. The patient exhibited symptoms of acute coronary syndrome, elevated troponins, and electrocardiographic changes consistent with STEMI. He was found to have significant coronary vasospasm on angiography that responded well to nitroglycerin. This case highlights the potential cardiovascular complications of COVID-19 infection, even when asymptomatic, and the importance of considering vasospasm as a possible mechanism in patients presenting with acute coronary syndrome. We also elaborate on some potential pathophysiological mechanisms in which COVID-19 may lead to coronary vasospasm.

## Introduction

Coronavirus 2019 (COVID-19) infected hundreds of millions and caused a worldwide pandemic [1]. Predominantly known as a respiratory virus, the effects of severe acute respiratory syndrome coronavirus 2 (SARS-CoV-2) spanned multiple organ systems affecting patients acutely and with long-term complications that are still not well understood. Amongst those complications is its effect on the cardiovascular system including myocarditis, pericarditis, ST-segment elevation myocardial infarction, stress-related cardiomyopathy, arrhythmias, spontaneous coronary artery dissection, thromboembolic complications, and coronary artery vasospasm (CAV) [2].

The virus’ effects on the endothelium have been proposed as the mechanism for its cardiovascular and thrombotic events. Other mechanisms include direct damage to myocytes via angiotensin-converting enzyme receptors resulting in damaged signaling pathways, myocardial ischemia via hypoxia, multiple microthrombi, systemic inflammatory response due to cytokine storm with vasculitis-like damage, and coronary spasms [3]. This has been supported by a paucity of reports of massive coronary thrombosis, venous thrombosis, type 1 and type 2 myocardial infarctions, as well as myocardial infarction with non-obstructive coronary arteries (MINOCA) [3]. In this report, we aim to display one of the potentially fatal complications of even asymptomatic COVID-19 infection, STEMI due to severe diffuse CAV. 

This article was previously presented as a poster abstract at the 2023 American College of Cardiology Annual Meeting on March 4, 2023.

## Case presentation

A 55-year-old male with a history of hypertension, anxiety, and gastroesophageal reflux disease was brought in by ambulance to the emergency department (ED) for chest pain. He was a current cigarette smoker of about one pack per day for 30 years. He was seen by his primary care doctor the day prior for a chief complaint of a headache and minor chest discomfort. At that time, an electrocardiogram (EKG) was said to be normal and an outpatient echocardiogram was ordered. The following day the chest pain worsened.

The pain was described as substernal, 8/10 in severity, and described as a burning sensation accompanied by diaphoresis and nausea which began that morning. The pain was nonreproducible, not positional, and not related to exertion or rest. Further review systems were unremarkable including no fevers, chills, cough, runny nose, vomiting, abdominal pain, diarrhea, or loss of taste or smell. He denied alcohol or drug use and his only medications were lisinopril and buspirone. Physical exam was unremarkable including cardiopulmonary exam.

**Figure 1:**
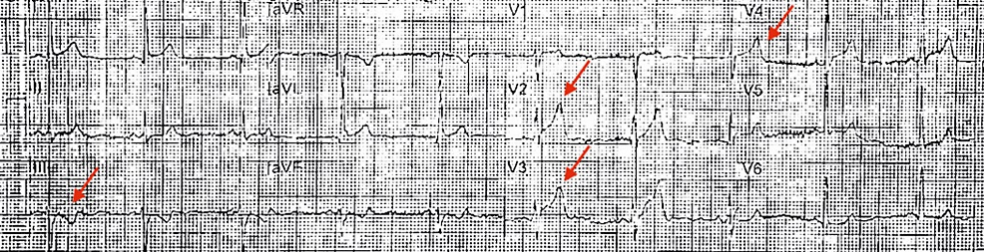
Initial EKG from the ambulance showing anterolateral 2mm ST elevations (arrows).

Initial EKG done by the paramedics in transport showed ST segment elevations in anterior leads and ST depressions inferiorly (Figure 1). He was given 325 mg aspirin and sublingual nitroglycerin which helped his pain. Upon arrival to the ED, blood pressure was 162/93 mmHg, heart rate was 89 beats per minute, respiratory rate was 16 per minute, and was saturating 99% on room air. 

He was started on a heparin drip, high-intensity statin, and a beta blocker. Initial labs showed a slight leukocytosis with normal differential and hemoglobin and platelet count. A complete metabolic panel revealed sodium of 140 mmol/L, blood urea nitrogen (BUN) 32 mg/dL (reference range 7-18 mg/dL), serum creatinine 1.44 mg/dL (reference range 0.55-1.3 mg/dL), aspartate aminotransferase (AST) 39 U/L (reference range 10-37 U/L), alanine aminotransferase (ALT) 153 U/L (reference range 12-78 U/L), and high sensitivity troponin of 1,325 ng/L (reference value <78 ng/dL) (Table 1).

**Table 1:** Laboratory values at admission.

Laboratory Test	Patient Result	Reference Range
White blood cell count (cells/microliter)	11,500	4,500 – 11,000
Hemoglobin (g/dL)	16.8	13.2 -16.6
Platelet (cells/microliter)	171,000	150,000- 450,000
Sodium (mmol/L)	140	135-145
Blood urea nitrogen (mg/dL)	32	7-18
Serum creatinine (mg/dL)	1.44	0.55-1.3
Aspartate aminotransferase (U/L)	39	10-37
Alanine aminotransferase (U/L)	153	12-78
High-sensitivity troponin-I (ng/L)	1,335	< 78
Repeat troponin-I (ng/L)	2,408	<78

**Figure 2:**
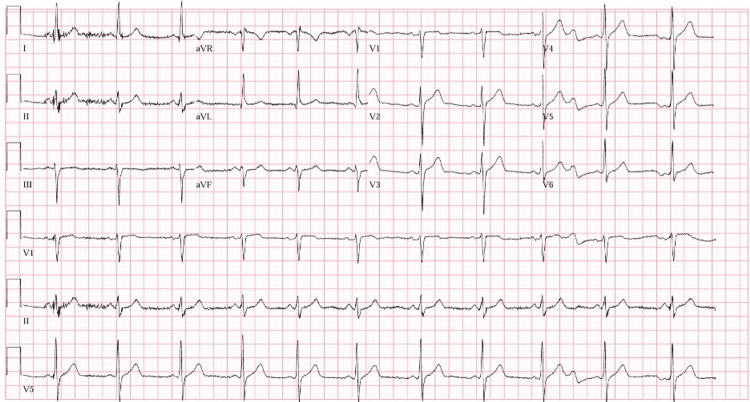
EKG in the ED after administration of sublingual nitroglycerin showing resolution of the ST segments elevations in lead V2-V4 that were previously seen on prior EKG.

Repeat EKG in the ED before being taken to the catheterization lab showed resolution of his ST elevations (Figure 2). Repeat high sensitivity troponin was 2,408 ng/dL. Per hospital policy, as this was during the COVID-19 pandemic, a COVID-19 rapid antigen swab was obtained and was positive, despite being currently asymptomatic and denying any previous symptoms leading up to this admission. Chest x-ray was clear without consolidation or infiltrates.

**Figure 3:**
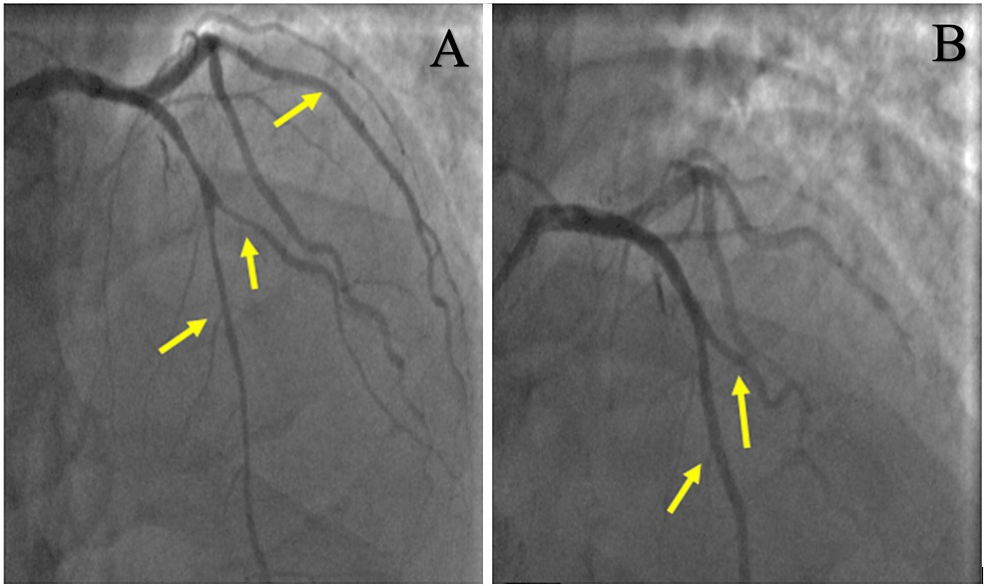
Coronary angiography showing diffuse coronary vasospasms in left anterior descending and left circumflex (arrows) before (A) and after (B) intra-coronary nitroglycerin administration.

Emergent coronary angiography (Figure 3) revealed smooth stenoses resulting in 50% proximal left anterior descending stenosis, 70% proximal left circumflex stenosis, and 30-50% right coronary artery stenosis with thrombolysis in myocardial infarction (TIMI) 1 flow and associated unstable ventricular tachycardia (VT) and anterolateral ST-elevations. Nitroglycerin was injected into the coronaries with an improvement of the coronary vasospasms, ventricular tachycardia, and ST-elevations. No wall motion abnormalities were noted once the vasospasms resolved, and no stents were placed since these findings were attributed to diffuse CAV. The patient was placed on a nitroglycerin drip and later transitioned to long-acting oral nitrates, beta-blocker, aspirin, and statin. He remained asymptomatic with no recurrence of chest pain after three months despite poor blood pressure control and heavy smoking.

## Discussion

Patients with COVID-19 infection frequently experience cardiovascular complications, affecting approximately 17-36% of patients [4]. The case of an asymptomatic COVID-19 patient presenting with STEMI due to CAV is both intriguing and challenging. This scenario highlights several important considerations regarding the pathophysiological mechanisms of COVID-19 and its cardiovascular complications, diagnostic challenges, and therapeutic implications. The mechanism by which COVID-19 may cause CAV is not fully understood, but it is thought to be related to the release of pro-inflammatory cytokines such as interleukin 6 and endothelial injury via angiotensin-converting enzyme receptors on the endothelial surface [4-6]. Furthermore, COVID-19 can infect endothelial cells, leading to endothelial dysfunction and subsequent vasoconstriction [5]. This can precipitate CAV, especially in patients with underlying endothelial damage or predisposition to vasospastic angina. Additionally, COVID-19 has been associated with autonomic dysfunction, which could also enhance sympathetic activity and precipitate CAV.

The diagnosis of CAV in the context of COVID-19, particularly when asymptomatic, poses significant challenges. In this case, the absence of typical respiratory symptoms of COVID-19 may lead to an initial underestimation of the role of the virus in the cardiac event. Initial triage with EKG can aid in the differential diagnosis of chest pain as COVID-19 has been known to cause pericarditis, STEMI, myocarditis, and spontaneous coronary artery dissection [5,7-8]. The EKG is aided by biomarkers such as high-sensitivity troponins in aiding in the triage of such patients. Confirming CAV can be done utilizing intracoronary acetylcholine which would induce the vasospasm, as did Azuma et al. [4]. Other modalities such as cardiac magnetic imaging can be used to assess the degree of myocardial damage as well as assess for other potential pathology as COVID-19 has demonstrated the vast array of diseases it can cause.

The therapeutic implications of managing STEMI in the context of CAV, especially in COVID-19-positive patients require a nuanced approach. In the acute setting, standard protocols for STEMI remain including anti-platelet agents, anti-coagulation, and reperfusion strategies such as coronary angiography [5]. In this case, vasospasm was found during angiography that resulted in significantly reduced flow, noted as TIMI 1 flow, as well as resulting in VT, which all resolved with intracoronary nitroglycerin. Long-term management includes addressing the COVID-19 infection as well as utilizing anti-anginal medications such as calcium channel blockers and long-acting nitrates [5]. In the context of COVID-19, the treatment regimen must also address the underlying inflammatory and prothrombotic states. Anti-inflammatory treatments, such as corticosteroids or other immunomodulatory agents, might be beneficial, although it has not been studied in this patient population [5].

Additionally, the asymptomatic nature of the COVID-19 infection in this patient is significant. It highlights the necessity for a high index of suspicion for COVID-19 in patients presenting with cardiovascular events, even in the absence of respiratory symptoms. Routine screening for COVID-19 in patients with unexplained cardiovascular events might be warranted, especially in areas with high community transmission.

## Conclusions

COVID-19 may have a wide range of cardiac involvement that may be difficult to discern on initial triage. One of these can be severe widespread coronary vasospasms even in asymptomatic COVID-19 infection, even in the absence of underlying coronary artery disease. Clinicians must be aware that even asymptomatic carriers of COVID-19 may still carry the pro-inflammatory and hypercoagulable effects of acute COVID-19 infection. 
